# Computer- und Magnetresonanztomographie in der Herzdiagnostik – welche Modalität ist die richtige? Eine Orientierungshilfe auf Basis der neuen Leitlinien der European Society of Cardiology (ESC)

**DOI:** 10.1007/s00117-022-01066-8

**Published:** 2022-10-12

**Authors:** Robin F. Gohmann, Malte M. Sieren, Matthias Gutberlet

**Affiliations:** 1 Abteilung für Diagnostische und, Interventionelle Radiologie, Herzzentrum Leipzig GmbH, Strümpellstr. 39, 04289 Leipzig, Deutschland; 2 Klinik für Radiologie und Nuklearmedizin, Universitätsklinikum Schleswig-Holstein, Campus Lübeck, Lübeck, Deutschland

**Keywords:** Computertomographie, Koronarangiographie, Kardiale Magnetresonanztomographie, Koronare Herzkrankheit, Brustschmerzen, Computed tomography, Coronary angiography, Cardiac magnetic resonance imaging, Coronary artery disease, Chest pain

## Abstract

In den Leitlinienaktualisierungen der European Society of Cardiology (ESC) nimmt die nichtinvasive radiologische Schnittbildgebung eine zunehmende prominente Rolle ein, während gleichzeitig die invasive Diagnostik weiter zurückgedrängt wird. Gerade für die Diagnose und die Behandlung des chronischen und des akuten Koronarsyndroms ergeben sich für die klinische Routine grundlegende Änderungen. Darüber hinaus bietet die Schnittbildgebung auch bei anderen kardialen Pathologien eine Alternative zur gängigen Primärdiagnostik, insbesondere der Echokardiographie, welche auch vermehrt in der Differenzialdiagnostik kardialer Erkrankungen eingesetzt wird. Der Radiologe sollte die Empfehlungen der aktuellen Leitlinien kennen und sich für ihre Etablierung im klinischen Alltag einsetzen. Diese Arbeit bietet eine Zusammenfassung der Indikationen kardialer Schnittbildgebung mit Fokus auf Neuerungen in den ESC-Leitlinien und geht auf typische Stärken und Schwächen der jeweiligen Modalität ein.

## Abstract (en)

In the guideline updates of the European Society of Cardiology (ESC), noninvasive radiological cross-sectional imaging is taking an increasingly prominent role, while at the same time invasive diagnostic approaches are becoming less important. Especially for the diagnosis and treatment of chronic and acute coronary syndromes, there are fundamental changes in clinical routine. In addition, cross-sectional imaging also offers an alternative to diagnostic algorithms for other cardiac pathologies, especially echocardiography, which is increasingly used in the differential diagnosis of cardiac diseases. The radiologist should be aware of the recommendations of the current guidelines and encourage their establishment in clinical practice. This paper summarizes the indications of cross-sectional cardiac imaging with focus on new recommendations in the ESC guidelines and addresses specific strengths and weaknesses of each modality.

In der letzten Dekade ist es zu einem Paradigmenwechsel in der kardiovaskulären Diagnostik gekommen. Durch die konsequente Translation wissenschaftlicher Evidenz in klinische Leitlinien wurde die radiologische, nichtinvasive Schnittbildgebung Schritt für Schritt zu einem Eckpfeiler der kardiovaskulären Diagnostik aufgewertet. Die kardiale Computer- und Magnetresonanztomographie (CT und MRT) stellen nach den aktuellen Leitlinien bereits heute die Methoden der ersten Wahl zur Primärdiagnostik kardialer Erkrankungen dar, ergänzen regelhaft die Basisdiagnostik oder sind gleichwertige Alternativen zu etablierten Methoden. Der Einsatz dieser Modalitäten im frühen Krankheitsstadium erlaubt die zielgerichtete und zeitnahe Evaluation der Pathologie – anatomisch wie funktionell – und bestimmt die weitere Behandlung des Patienten [10]. Die Bereitstellung, Durchführung und Beurteilung dieser Untersuchungen verlangt vom Radiologen sowohl die genaue Kenntnis der Indikation, Fachkenntnis der Pathologien als auch eine suffiziente interdisziplinäre Kommunikation mit den beteiligten Fachdisziplinen, um eine leitliniengerechte Behandlung des Patienten zu gewährleisten und Neuerungen im klinischen Alltag zu etablieren.

Dieser Artikel soll eine erste Orientierungshilfe zu den Stärken der jeweiligen Modalität und den möglichen Einsatzfeldern geben. Ein besonderer Fokus liegt auf den Aktualisierungen in kürzlich veröffentlichen Leitlinien der European Society of Cardiology (ESC).

## Aktualisierte Leitlinien

Richtungsweisende Neuerungen in den aktuellen Leitlinien finden sich vor allem in den Empfehlungen zur Diagnostik des breiten Spektrums ischämischer Herzerkrankungen: des akuten (ACS) und des chronischen Koronarsyndroms (CCS), das den Begriff der stabilen und chronischen koronaren Herzkrankheit in den ESC Leitlinien von 2019 ersetzt hat. Hier wurden von der ESC für die Diagnose und Behandlung des akuten und des chronischen Koronarsyndroms im Jahr 2019 und 2020 aktualisierte Leitlinien veröffentlicht [7, 22, 31]. Auch von der American Heart Association (AHA) und dem American College of Radiology (ACR) gab es eine Aktualisierung der Empfehlungen zur Beurteilung und Diagnose von Brustschmerz [21]. Eine der Kernänderungen basiert u. a. auf den Erkenntnissen von zahlreichen, multizentrischen CT-Studien, wie der PROMISE- und SCOT-Heart-Studie [15, 21, 29, 39]. Die aus den Studiendaten ermittelten Vortestwahrscheinlichkeiten für das Vorliegen einer koronaren Herzerkrankung (KHK) waren deutlich niedriger als bisher durch das *Diamond and Forrester Model* [22] berechnet, sodass Patienten nun mehrheitlich einem niedrigen bis mittleren Risiko zugeordnet werden müssen und damit ein deutlich größeres Patientenkollektiv als bisher nichtinvasiv untersucht werden sollte. Diese Entwicklung geht zu Lasten der invasiven Herzkatheteruntersuchung (HKU), deren Indikation folglich deutlich enger gestellt werden muss. Die 2020 veröffentlichten Ergebnisse der ISCHEMIA-Studie zeigten außerdem, dass Patienten von einer initialen invasiven Diagnostik lediglich symptomatisch profitieren und sich keine Verbesserung bezüglich kardiovaskulärer ischämischer Ereignisse oder der Sterblichkeit ergeben, wenn eine Hauptstammstenose zuvor mittels CT-Koronarangiographie („coronary computed tomography angiography“, CCTA) ausgeschlossen wurde [35]. Insgesamt hat dies nicht nur zu einer prominenteren Rolle der Schnittbildgebung als Ganzes, sondern vor allem auch zu einer Stärkung der anatomisch-morphologischen Bildgebung mittels CT gegenüber der funktionellen MRT-Bildgebung bei ACS und CCS geführt.

Neben den Leitlinien zum ACS und CCS wurden sowohl von der ESC als auch der AHA/ACR die Leitlinien zur akuten und chronischen Herzinsuffizienz [25, 36], Klappenerkrankungen [38, 45] und Erwachsenen mit angeborenen Herzfehlern aktualisiert [4]. Während, mit Ausnahme der Bildgebung des CCS und verschiedener angeborener Herzfehler, die kardiale CT und MRT bei diesen Pathologien nicht zur Basisdiagnostik gehört, etabliert sie sich dennoch als alternative Untersuchungsmethoden zur Funktionsbeurteilung (MRT) und u. a. zur Umfelddiagnostik bzw. zum Ausschluss der koronaren Herzkrankheit (KHK) mittels CT und der Differenzialdiagnostik beim ACS mittels MRT.

## Stärken der Schnittbildgebung

### Computertomographie

Die generellen Vorteile der CT – flächendeckende, unmittelbare Verfügbarkeit und kurze Untersuchungsdauer [16, 41] – sind v. a. in der Notfalldiagnostik von entscheidender Bedeutung. Außerdem ist die CT für ein größeres Patientenkollektiv unmittelbar zugänglich, auch für solche mit metallischen Implantaten oder Klaustrophobie. Die Strahlenexposition hat sich in den letzten 10 Jahren um ca. die Hälfte verringert und liegt für eine CCTA aktuell im Median bei 4,54 mSv [16]. Gepaart mit der hohen räumlichen und inzwischen ausreichend hohen zeitlichen Auflösung, eignet sich die CT hervorragend zur anatomischen Beurteilung des Herzens und der Herzkranzgefäße (Abb. 2). Nichtsdestotrotz können Bewegungsartefakte bei erhöhter Herzfrequenz und Arrhythmien sowie Aufhärtungsartefakte durch Fremdmaterial oder hohe Kalklast die Untersuchungsqualität beeinträchtigen. Auch muss der Patient kooperativ sein und den Atem für 5–10 s anhalten können. In der Patientenselektion sollten diese Faktoren bedacht bzw. die Herzfrequenz im Vorfeld medikamentös mittels β‑Blockern auf eine Zielfrequenz von 60–70 Schlägen pro Minute eingestellt werden [24, 40]. Um Bewegungsartefakte zu reduzieren, gilt eine 64-Detektorzeilen-CT als Mindeststandard. Konkrete Protokollempfehlungen sind natürlich abhängig vom Gerätetyp und konkreten Patienten, Publikationen von Fachgesellschaften können jedoch Anhalt liefern [1, 5, 17].

**Figure 1:**
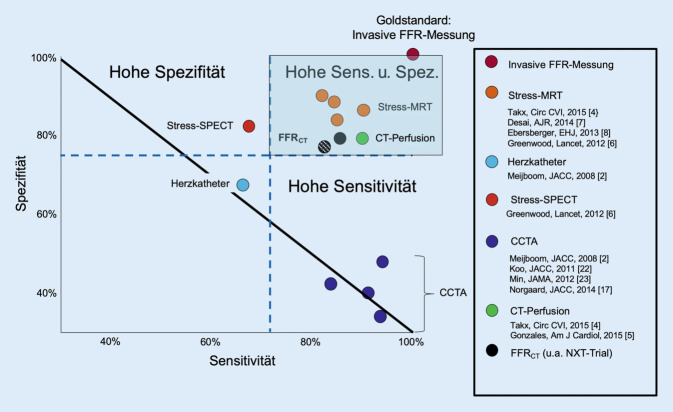

**Figure 2:**
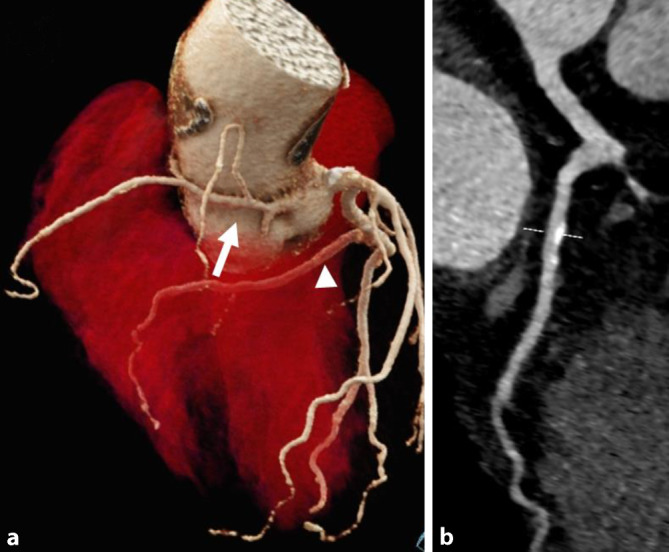

Die Koronarangiographie mittels CTA nimmt als robuste Methode – v. a. wegen ihrer hohen Sensitivität und ihres hohen negativ-prädiktiven Werts – eine Schlüsselrolle in der Diagnostik des CCS ein Abb. 1. Die CCTA ermöglicht sowohl die Diagnose einer stenosierenden und nichtstenosierenden KHK als auch von Koronaranomalien und ist hierbei sogar der HKU überlegen (Abb. 2). Neben klassischen Charakteristika wie Stenosegrad und -länge wurden in der PROMISE- und ROMICAT-II-Studie weiterführende Parameter, wie niedrige Plaquedichte und Remodeling für eine noch akkuratere Risikostratifizierung untersucht [11, 12]. Auch dies ist mit der invasiven HKU, die nur das Lumen darstellt, nicht in gleichem Maße möglich. Mit der CAD-RADS-Klassifikation liegt außerdem ein strukturiertes „decision support tool“ vor, das eine Standardisierung ermöglicht und die interdisziplinäre Kommunikation erleichtert [14]. Von der Arbeitsgemeinschaft Herz- und Gefäßdiagnostik der Deutschen Röntgengesellschaft wurden darüber hinaus Befundungsvorlagen basierend auf einem Experten- und Qualitätskonsensus konzipiert (www.befundung.drg.de; [6]).

**Figure 3:**
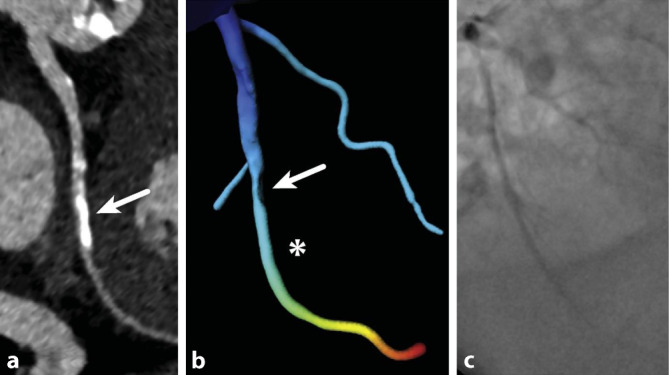

Eine native, dosisreduzierte („low-dose“) CT zur Beurteilung der Kalklast mittels des Agatston-Scores [3] erlaubt es zusätzlich, einen gut reproduzierbaren quantitativen Wert zur Beurteilung des kardiovaskulären Risikos zu bestimmen [22]. Eine extrem hohe Kalklast kann sich außerdem auf die Beurteilungsqualität der CCTA auswirken (Abb. 3) und den Untersucher veranlassen, funktionelle Untersuchungen, z. B. eine kardiale MRT (CMR) der CCTA, vorzuziehen. Zukünftig erlauben neue technische Entwicklungen, wie die geräteseitige Weiterentwicklung mit Dual-Energy, Spektral- oder Photon-Counting-Scannern, eine weitere Verbesserung der Bildqualität inklusive einer weiteren Reduktion von Artefakten [26, 32, 44], z. B. durch Kalk oder metallisches Fremdmaterial (v. a. Stents).

Neben dem klinisch etablierten Agatston-Score werden auch quantitative funktionelle Parameter beforscht. Hier gibt es vielversprechende Ergebnisse zur CT-basierten fraktionellen Flussreserve (CT-FFR; Abb. 3; [19]) – dem Pendant zur mittels invasiver Druckmessung im Herzkatheter bestimmten FFR [27]. Die FFR gibt das Verhältnis des mittleren arteriellen Drucks hinter einer Koronarstenose im Vergleich zum maximalen Druck vor der Stenose unter Adenosinbelastung an und wird mittlerweile als Goldstandard zur Ermittlung der hämodynamischen Relevanz einer Koronarstenose angesehen [23].

Die Durchführung einer CT-Perfusion ist ebenfalls mittels pharmakologischer Induktion möglich. Auch hier sind erste Ergebnisse vielversprechend, aber angesichts der Verfügbarkeit alternativer Tests (insbesondere der CMR, aber auch der Myokardszintigraphie und Stress-Echokardiographie) ist diese Untersuchung nur in Einzelfällen indiziert. Die europäischen Leitlinienempfehlungen zum Einsatz der CT beschränken sich aktuell auf die etablierte anatomisch-morphologische Bildgebung. In den aktuellen AHA-Leitlinien zum Thoraxschmerz von 2021 wird auch die ergänzende CT-FFR aufgeführt, deren Bestimmung in den USA auch vergütet wird.

### Magnetresonanztomographie

Die kardiale Magnetresonanztomographie gehört mit zu den aufwändigsten bildgebenden radiologischen Untersuchungen in Bezug auf Zeit und Durchführung. Obwohl im direkten Vergleich weniger breit verfügbar und durch längere Untersuchungsdauer weniger gut für die Notfallversorgung geeignet, hat sich in Deutschland ein flächendeckendes Netz aus zertifizierten Zentren und Experten etabliert [41]. Dementsprechend werden Patienten mit der CMR häufig in einem elektiven Setting und nach Durchführung von Basisdiagnostik untersucht.

**Figure 4:**
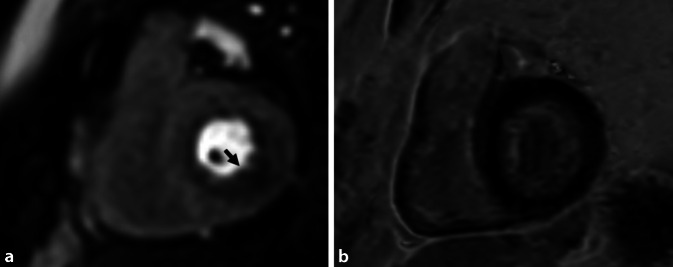

**Figure 5:**
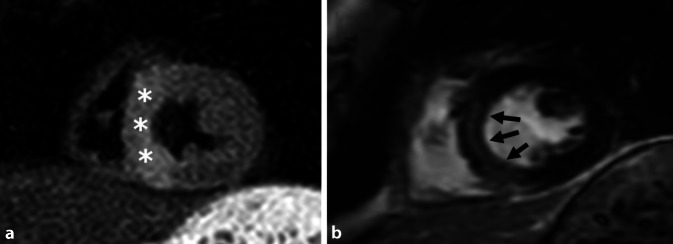

**Figure 6:**
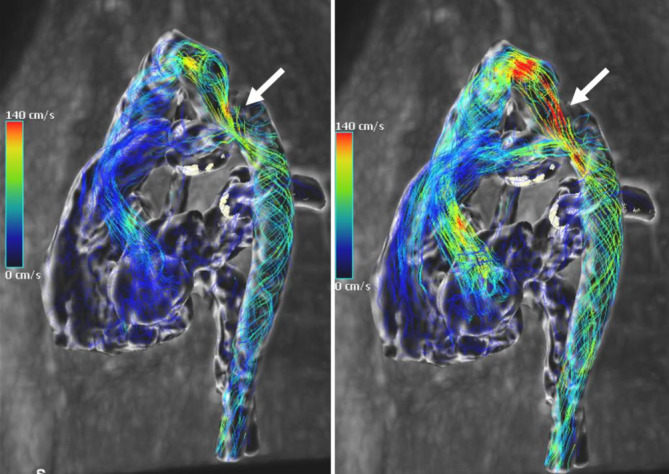

Die Stärken der MRT liegen bezüglich der KHK-Diagnostik vor allem in der funktionellen Beurteilung der Herzperfusion (Abb. 4), der Differenzierung von akut und chronisch verändertem Myokard (Abb. 5) sowie der dynamischen Beurteilung der Funktion (Abb. 6). Hierdurch resultiert eine erhöhte Spezifität im Vergleich zu morphologischen Verfahren (CT, HKU) für das Vorliegen einer hämodynamisch relevanten Koronarstenose (Abb. 4), entzündlicher und fibrosierender Myokardveränderungen (Abb. 5) sowie den hiermit assoziierten Pathologien. Des Weiteren liefert die CMR Informationen über Kinetik und Blutfluss mit der Möglichkeit zur Quantifizierung dieser (Abb. 6). Alternative, auf funktionale Aspekte fokussierte Untersuchungsmodalitäten sind die Single-Photon-Emissions-Computertomographie (SPECT), die Positronen-Emissions-Tomographie(PET)/CT und die Stress-Echokardiographie, welche jedoch einzeln nicht alle Informationen der MRT liefern können.

Auch für die CMR sind technischen Anforderungen und Untersuchungsprotokolle zunehmend standardisiert, und es liegen Empfehlungen entsprechender Fachgesellschaften vor [33]. Moderne 1,5- und 3‑T-MRT-Scanner – wie sie in radiologischen Institutionen vorgehalten werden – erlauben in der Regel die Durchführung von CMR-Untersuchungen. Gleichzeitig ist eine Vielzahl medizinischer Implantate, insbesondere orthopädische Prothesen und Herzschrittmacher, inzwischen bedingt oder vollständig MRT-sicher (*MR-conditional; MR-safe*). So kann auch dieses Patientenkollektiv nach entsprechender Vorbereitung und Befolgung der spezifischen Herstellerangaben einer MRT-Untersuchung zugeführt werden [42]. Auch für die MRT liegen unterstützende Dokumente zu Befundungsstandards von 2009, welche aktuell überarbeitet werden [28], sowie strukturierte Befundungstemplates der Arbeitsgemeinschaft Herz- und Gefäßdiagnostik der DRG vor [6].

## Leitsymptome

Anhand typischer Leitsymptome werden im Folgenden Anwendungsbeispiele für die kardiale CT und MRT vorgestellt. Der Fokus liegt dabei auf sehr häufigen Pathologien, zu denen eine Aktualisierung der Leitlinien stattgefunden hat.

### Stabiler/chronischer Brustschmerz

Die stabile Angina pectoris ist das Leitsymptom des chronischen Koronarsyndroms („chronic coronary syndrome“, CCS). Die Symptome bei Angina pectoris sind in der Regel im Brustkorb in der Nähe des Brustbeins lokalisiert, können aber überall vom Epigastrium, bis zum Unterkiefer oder im Arm auftreten. Sie werden häufig als „Brennen“ oder „Engegefühl“ beschrieben, dauern in den meisten Fällen weniger als 10 min an und sind mit körperlicher Belastung assoziiert. Klinisch ist die Unterscheidung zwischen stabiler und instabiler Angina essenziell und für das weitere Procedere entscheidend [31]. Viele Patienten mit einem CCS und demzufolge nun stabiler Angina pectoris hatten in der Vergangenheit eine Phase mit instabiler Angina [31].

Die CCTA hat unter den nichtinvasiven Tests die höchste Sensitivität und v. a. den höchsten negativ-prädiktiven Wert zum Ausschluss einer obstruktiven koronaren Herzerkrankung (Abb. 1). In der klinischen Routine werden in der Regel noch eine Echokardiographie und ein Ruhe-EKG vorgeschaltet. Die Auswahl des geeigneten diagnostischen Verfahrens hängt im Wesentlichen von der Vortestwahrscheinlichkeit ab, die anhand von Alter, Geschlecht und Charakter der Beschwerden (Tab. 1) abgeschätzt werden kann [31].

**Table 1:** 

Typische Angina	Erfüllt die folgenden drei Merkmale:
	1. Beklemmende Beschwerden im vorderen Teil der Brust oder in Hals, Kiefer, Schulter oder Arm
	2. Ausgelöst durch körperliche Anstrengung
	3. Linderung durch Ruhe oder Nitrate innerhalb von 5 min
Atypische Angina	Erfüllt zwei der genannten Merkmale
Nichtanginöse Brustschmerzen	Erfüllt nur eines oder keines der genannten Merkmale

Der notwendige Schwellenwert einer *Vortestwahrscheinlichkeit von >* *85* *% (hohes Risiko), *um primär eine invasive HKU erwägen zu können, wird nach der Aktualisierung nur noch selten erreicht, sodass in aller Regel die nichtinvasive Bildgebung für die Primärdiagnostik der KHK indiziert ist. Die HKU wird nur noch als Alternative zur nichtinvasiven Primärdiagnostik bei hoher klinischer Vortestwahrscheinlichkeit empfohlen, die kaum noch einem Patienten mit stabilem CCS zuzuordnen ist. Bei Patienten mit einer *Vortestwahrscheinlichkeit von 15–85* *% (mittleres Risiko) *werden laut den ESC-Leitlinien von 2019 zur Primärdiagnostik einer stabilen KHK sowohl die CCTA als morphologisches Verfahren oder funktionelle Verfahren (Stress-CMR, Stress-Echokardiographie, Myokardszintigraphie) empfohlen. Die CCTA sollte hierbei v. a. für die Patienten mit niedriger bis mittlerer intermediärer Vortestwahrscheinlichkeit (< 50 %) eingesetzt werden und die funktionellen Verfahren eher für die Patienten mit höherer klinischer Vortestwahrscheinlichkeit (> 50 %), die nach den neu ermittelten Vortestwahrscheinlichkeiten ebenfalls nur noch von wenigen Patienten erreicht werden [22].

Die Auswahl der Modalität sollte weiterhin von der lokalen Expertise bzw. der erwarteten patientenspezifischen Untersuchungsqualität abhängen. Bei Patienten mit unklarer Signifikanz der KHK nach einer CCTA sollte ein funktionelles Verfahren, z. B. eine CMR oder Myokardszintigraphie, durchgeführt werden.

Selbst bei Patienten mit einer *Vortestwahrscheinlichkeit von 5–15* *% (geringes Risiko) *kann eine nichtinvasive Diagnostik nach den neuen Leitlinien indiziert sein. Zu den bekannten Berechnungsfaktoren wie Alter, Geschlecht und Beschwerdesymptomatik werden dann zusätzlich noch die kardialen Risikofaktoren (arterielle Hypertonie, Diabetes mellitus, Hyperlipoproteinämie, Nikotinabusus oder eine positive Familienanamnese) und die Ergebnisse bereits durchgeführter Voruntersuchungen (z. B. Echokardiographie, Belastungs-EKG oder auch ein Kalziumscoring) mitberücksichtigt, um die sog. *klinische Wahrscheinlichkeit* zu ermitteln. Sind diese Faktoren vorhanden, erhöht sich die klinische Wahrscheinlichkeit entsprechend. Die Stärke der CCTA liegt vor allem im Ausschluss einer KHK, weshalb sie insbesondere bei Patienten mit niedrigem bis mittlerem Risiko bevorzugt eingesetzt werden sollte. Auch bei jungen Patienten übertrifft der Nutzen einer korrekten Diagnose die mit der Strahlenexposition einhergehenden Risiken [22, 24, 31]. Bei Patienten mit einer *Vortestwahrscheinlichkeit von <* *5* *% (sehr geringes Risiko)* sollte hingegen keine weitere Diagnostik durchgeführt werden [31].

Der Stellenwert des Kalziumscorings bei symptomatischen Patienten ist unklar. Weder die oft postulierte* Gatekeeper-Funktion* noch die fehlende diagnostische Aussagekraft der CCTA bei hoher Kalklast lassen sich zweifelsfrei belegen [18–20]. Die Leitlinie empfiehlt zwar bei hoher Kalklast eher von der CCTA abzusehen [31], doch kann auch bei einem sehr hohen Kalziumscore (> 1000) der Ausschluss einer obstruktiven KHK noch immer möglich sein [18]. Eine native CT zum Kalziumscoring kann dennoch durchgeführt werden, um einen zusätzlichen Risikoparameter zu generieren und ggf. eine frühzeitige Indikation für eine funktionelle Untersuchung, z. B. eine Stress-CMR, zu stellen. Auch kann anhand des Kalziumscorings eine präzisere Planung der CCTA erfolgen und so eine Dosisersparnis von bis zu 16 % erzielt werden [34].

### Akuter Brustschmerz

Der akute Brustschmerz ist eines der häufigsten in der Akutmedizin anzutreffenden Symptome und hinweisend für das Vorliegen eines akuten Koronarsyndroms (ACS). Unter dem Sammelbegriff ACS werden der Herzstillstand, elektrische oder hämodynamische Instabilität mit kardiogenem Schock aufgrund anhaltender Ischämie oder mechanischer Komplikationen wie einer schweren Mitralinsuffizienz, bis hin zu Patienten, die zum Zeitpunkt der Vorstellung bereits wieder schmerzfrei sind, zusammengefasst. Etwa 50 % der Patienten mit akutem Brustschmerz leiden an einer primär kardialen Pathologie. Die anderen 50 % der Patienten leiden an extrakardialen Ursachen, wie der Lungenarterienembolie oder einer akuten Aortendissektion, die gut mit der CT diagnostiziert werden können. Die Abgrenzung zur stabilen Angina pectoris kann u. a. durch folgende Charakteristika erfolgen:

- länger andauernde (> 20 min) Brustbeschwerden in Ruhe oder minimaler Belastung,
- eine neu aufgetretene (de novo) Angina pectoris (< 3 Monate),
- eine Symptomverschlechterung einer zuvor stabilen Angina pectoris,
- eine Angina pectoris nach Myokardinfarkt [7].

Wichtige Differenzialdiagnosen umfassen, wie schon erwähnt, die Lungenarterienembolie, die Aortendissektion und den Spannungspneumothorax [7], aber auch die akute Myokarditis, die Sarkoidose und die Tako-Tsubo-Kardiomyopathie – letztere können gut mit der CMR diagnostiziert werden.

Die Rolle der Bildgebung liegt beim akuten Brustschmerz v. a. in der Sicherung der Diagnose nach erfolgter Basisdiagnostik und daraus gefolgertem Verdacht auf eine kardiovaskuläre Erkrankung. Beim ST-Hebungsinfarkt ist weiterhin eine unmittelbare Rekanalisierung mittels Herzkatheter indiziert. Bei Patienten mit niedriger bis mittlerer klinischer Wahrscheinlichkeit bezüglich des Vorliegens einer KHK und unauffälligem bzw. nicht eineindeutigem Troponin- und/oder EKG-Befund wird nun erstmalig auch die CCTA als alternatives Untersuchungsverfahren zur HKU empfohlen. Die CCTA hat einen hohen negativen Vorhersagewert für den Ausschluss einer KHK und damit für das Vorliegen eines ACS [7, 21]. Dementsprechend ist die CCTA weniger nützlich bei Patienten, bei denen bereits eine KHK bekannt ist [7]. Darüber hinaus ist der Einsatz der CCTA in der Akutsituation bei Patienten mit Stents oder einer vorangegangenen koronaren Bypassoperation bisher nicht ausreichend wissenschaftlich validiert. Für den Einsatz der CCTA im akuten Setting spricht, dass auch die oben genannten, potenziell letalen und therapeutisch zeitkritischen Differenzialdiagnosen ausgeschlossen werden können.

Der Zeitfaktor ist auch die wesentliche Einschränkung beim Einsatz der CMR in der Akutsituation. Obwohl die kardiale MRT einen Myokardinfarkt mit hoher Sensitivität und Spezifität direkt nachweisen kann, spielt sie im akuten Notfallsetting, auch bedingt durch die unmittelbare Verfügbarkeit der CT, eine untergeordnete Rolle. Einsatzfelder der CMR sind etwa die Vitalitätsbeurteilung vor Koronarintervention [30] oder nach erfolgter Koronarintervention zur Risikostratifizierung [8], der Nachweis einer „culprit lesion“ oder der Myokardinfarkt ohne obstruktive Koronare Herzerkrankung („myocardial infarction with nonobstructive coronary arteries“, MINOCA; [9]). Bei Letzterem ist die CMR eines der wichtigsten Diagnoseinstrumente, da sie bei mehr als 85 % der Patienten die zugrundeliegende Ursache identifiziert [7, 21], wie z. B. eine akute Myokarditis, eine kardiale Sarkoidose, ein Tako-Tsubo-Kardiomyopathie oder eben eine MINOCA.

### Leistungsminderung

Unter das unspezifische Symptom Leistungsminderung fällt ein breites Spektrum an Herzerkrankungen und nichtkardialen Pathologien. Dementsprechend ist hier eine klinische Evaluation inklusive Anamnese und einer Basisdiagnostik vor Durchführung einer radiologischen Schnittbildgebung besonders wichtig. Die geäußerte Verdachtsdiagnose bestimmt dann wie auch in anderen Szenarien die Auswahl der Modalität und des Untersuchungsprotokolls.

Die klinische Präsentation von Patienten mit Myokarditis ist sehr variabel. Sie kann von chronischer Fatigue mit Leistungsminderung, bis hin zu akutem Brustschmerz mit Symptomen einer Herzinsuffizienz und erhöhten Troponinwerten reichen – dann ist die Myokarditis eine Differenzialdiagnose zum ACS. Bei Verdacht auf eine Myokarditis ist die CMR das diagnostische Mittel der ersten Wahl. Der Einsatz von Mapping zum Nachweis einer Myokardentzündung wird in diesem Zusammenhang dringend empfohlen, da es sich positiv auf die diagnostische Genauigkeit, insbesondere auch die Intra- und Interobservervariabilität, auswirkt [13, 46]. In ähnlicher Weise kann die CMR für die Identifizierung verschiedener chronischer Entzündungszustände – von chronischer Myokarditis, über Sarkoidose bis hin zu HIV-assoziierten myokardialen Erkrankung – zum Ausschluss eines Entzündungsfokus als Ursprung für aufgetretene Arrhythmien nützlich sein.

**Figure 7:**
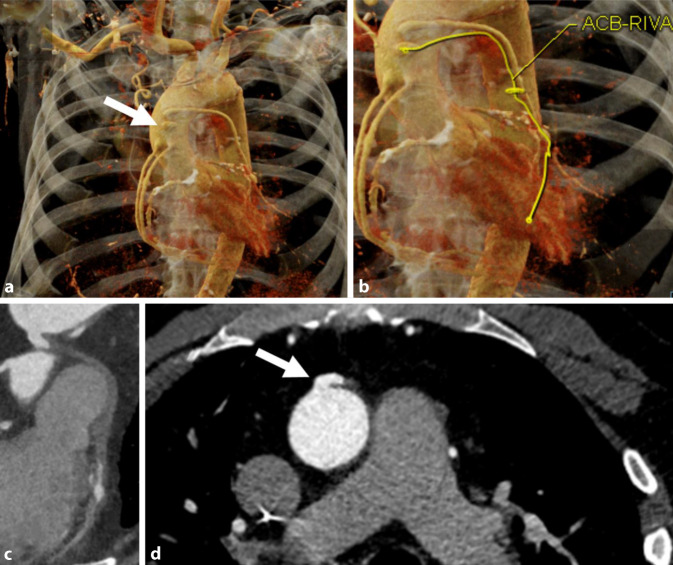

Die Basisdiagnostik für Patienten mit V. a. auf eine Herzklappenstenose oder -insuffizienz ist unverändert die Echokardiographie. Sind die Ergebnisse der Echokardiographie inkonklusiv, ist die CMR die Methode der Wahl, um den Schweregrad von Herzklappenläsionen und insbesondere die Regurgitationsfraktion zu bestimmen. Die erhobenen Funktionsparameter können entscheidend für die Indikationen einer operativen Therapie sein. Darüber hinaus stellt die CMR den Goldstandard für die Funktionsbeurteilung des Ventrikelvolumens, insbesondere des rechten Ventrikels und etwaiger Myokardfibrosen, dar. Die Rolle der CT fokussiert sich primär auf die Umfelddiagnostik. Diese sollte durchgeführt werden, wenn in der Echokardiographie ein Aortendiameter von > 40 mm vermutet wird. Für die Planung von Transkatheter-Aortenklappen-Implantation (TAVI) ist sie unerlässlich (Abb. 7). Außerdem wird sie als Minorkriterium in der Diagnose einer hochgradigen Aortenklappenstenose (Kalziumscoring) und zur Diagnosesicherung/Verlaufsbeurteilung einer Thrombose der Klappenprothese eingesetzt. Des Weiteren empfehlen sowohl die ESC- als auch die ACC/AHA-Leitlinien die CCTA zum Ausschluss einer KHK vor Klappeninterventionen [38, 45].

Bei Patienten mit Verdacht auf eine Herzinsuffizienz sollten in der Primärdiagnostik eine transthorakale Echokardiographie und ein Röntgenbild des Thorax durchgeführt werden. Eine kardiale MRT wird bei Patienten mit schlechtem akustischem Fenster in der Echokardiographie und bei Verdacht auf eine zugrundeliegende Kardiomyopathie, Hämochromatose, Amyloidose oder Myokarditis empfohlen. Generell wird die CMR bei Verdacht auf eine nichtischämische Kardiomyopathie zur Sicherung der Diagnose, Einordnung der Entität und zur Funktionsbeurteilung des Myokards eingesetzt (Abb. 4 und 3). Die CCTA kann wiederum auch in diesem Kontext zum Ausschluss einer koronaren Herzkrankheit herangezogen werden [25, 36].

## Weitere Anwendungsgebiete

Weitere Indikationen für kardiale Schnittbildgebung sind die Evaluation von Perikarderkrankungen und intrakardialen Thromben. Die ESC-Leitlinien für Perikarderkrankungen von 2015 empfehlen nachdrücklich die CT und/oder die CMR als zweite Stufe der diagnostischen Abklärung bei einer Perikarditis [2].

Kardiale Thromben werden regelhaft mittels transthorakaler oder transösophagealer Echokardiographie untersucht, die Sensitivität und Spezifität der CMR ist aber als gleichwertig anzusehen [43] und nur sinnvoll, wenn echokardiographisch nicht beurteilbar.

Die Betreuung von Patienten mit angeborenen Herzfehlern sollte in aller Regel an Zentren mit entsprechender Expertise in Diagnostik und Therapie erfolgen. Auch bei diesen Patienten stellen die Echokardiographie und das Röntgenbild des Thorax die Basis der bildgebenden Diagnostik dar. Die CMR hat eine zentrale Bedeutung für die zuverlässige Bestimmung und Verlaufsbeurteilung von Ventrikelvolumina, Auswurfleistung, systemischem und pulmonalem Blutfluss sowie von Shuntvolumina. Die CT als primär morphologisches Verfahren wird in der Regel für spezifischere Indikationen, welche die Morphologie betreffen, insbesondere postoperativ und in der Notfalldiagnostik herangezogen und ist natürlich auch in dieser Patientengruppe besonders geeignet für die Beurteilung koronararterieller Pathologien (Abb. 2) und die präzise Visualisierung von aortopulmonalen Kollateralen [4].

## Fazit für die Praxis

- Die Aktualisierung der Leitlinien zur Diagnostik und Therapie des akuten und chronischen Koronarsyndroms definieren die kardiale Computertomographie (CT) und Magnetresonanztomographie (MRT) als hochsensitive und -spezifische Schlüsselmodalitäten.
- Die CT-Koronarangiographie (CCTA) – häufig gepaart mit nativem Kalziumscoring – ist sowohl in der Akut- als auch Elektivmedizin ein verlässlicher Eckpfeiler des diagnostischen Algorithmus der koronaren Herzkrankheit (KHK). Sie ist bei stabiler Angina pectoris als auch bei akutem Brustschmerz ohne ST-Hebung in den meisten Fällen die Modalität der ersten Wahl („first line“) und dient als *Gatekeeper* für die funktionellen nichtinvasiven Verfahren.
- Die Stärke der MRT ist die Funktionsbeurteilung des Myokards und die Differenzierung von Myokardveränderungen. Sie nimmt bei der KHK die Rolle einer *Second-line*-Untersuchungsmethode, z. B. bei eingeschränkter Beurteilbarkeit der CCTA aufgrund hoher Kalklast, bzw. als ergänzende Untersuchungsmethode zur Differenzialdiagnose beim Ausschluss einer KHK ein.
- Die MRT ist die First-line-Untersuchungsmethode bei Pathologien aus dem Formenkreis der entzündlichen Herzerkrankungen, der Kardiomyopathien und von zentraler Bedeutung bei angeborenen Herzfehlern.
